# Evaluation of the Flexural Strength and Translucency of Zirconia With Different Microstructures

**DOI:** 10.7759/cureus.95769

**Published:** 2025-10-30

**Authors:** Merve Buse Kultas Kaleli

**Affiliations:** 1 Prosthodontics, Meram Oral and Dental Health Center, Konya, TUR

**Keywords:** flexural strength, monolithic zirconia, multilayer zirconia, spectrophotometry, translucency

## Abstract

Objective: This study aimed to evaluate the translucency and flexural strength (FS) of three zirconia ceramics with different microstructural characteristics and to examine the relationship between these properties.

Materials and methods: Zirconia specimens were prepared from IPS e.max ZirCAD Prime A2 (Ivoclar Vivadent, Schaan, Liechtenstein), Vita YZ HT (Vita Zahnfabrik, Bad Säckingen, Germany), and IPS e.max ZirCAD MT Multi A2 (Ivoclar Vivadent) blocks. A total of 30 specimens were fabricated using CAD/CAM technology with a 20% sintering shrinkage adjustment. Translucency was measured using a spectrophotometer based on the CIE L*a*b* system (Commission Internationale de l'Éclairage, Vienna, Austria), and the translucency parameter (TP) was calculated using the CIEDE2000 formula. FS was determined using a three-point bending test according to ISO 6872 standards. Statistical analysis was performed using the Kruskal-Wallis and Spearman correlation tests (p < 0.05).

Results: Although IPS e.max ZirCAD MT exhibited the highest translucency (p > 0.05). and Vita YZ HT showed the highest FS (p<0.05). No significant correlation was found between translucency and FS in any group.

Conclusion: Within the limitations of this in vitro study, zirconia ceramics with different microstructures showed no statistically significant differences in translucency but statistically significant differences in FS.

## Introduction

Zirconia is commonly preferred in prosthetic restorations due to its excellent chemical stability, high mechanical strength, and dimensional reliability [1]. Zirconia exhibits polymorphism at different temperatures, existing in three allotropic phases: monoclinic (room temperature to 1170°C), tetragonal (1170°C-2360°C), and cubic (2360°C up to its melting point at 2680°C). The tetragonal and cubic phases can be preserved at room temperature by stabilization with various cubic oxides, such as yttrium oxide (Y₂O₃), calcium oxide (CaO), and magnesium oxide (MgO) [2,3]. Polycrystalline ceramics are characterized by fine grains that provide high strength and fracture toughness, but their translucency remains limited. Increasing the yttria content of zirconia above 3 mol% enhances translucency by partially stabilizing the cubic phase; however, this improvement compromises mechanical strength in 5 mol% yttria partially stabilized zirconia (5Y-PSZ) [4,5]. Despite this reduction, these modified zirconia ceramics still exhibit higher strength compared to alternative materials, such as lithium disilicate glass ceramics [5].

The improvement in translucency of zirconia ceramics has facilitated their use in esthetically pleasing monolithic restorations [6]. Highly translucent yttria-stabilized zirconia (YSZ) has attracted attention for its superior optical properties over conventional 3Y-TZP. Incorporating 4 mol% or more yttria into zirconia increases cubic phase content, thereby significantly enhancing translucency [7]. Although the increase in the optically isotropic cubic phase enhances translucency, it leads to a reduction in mechanical properties compared to previous generations [8,9]. Zirconias with higher cubic phase content offer greater translucency while maintaining similar elastic modulus and hardness; however, they exhibit lower flexural strength (FS), fracture toughness, and fatigue resistance [9-11].

Color evaluation is a subjective process influenced by factors such as the object’s and observer’s position relative to light and the observer’s emotional state. This subjectivity can be reduced by using a spectrophotometer, which measures the three color components (L*, a*, b*) and also assesses translucency. Depending on light scattering, ceramics may appear opaque or translucent [12].

Zirconia is an oxide ceramic characterized by a high fracture toughness ranging from 5 to 10 megapascals (MPa) and a FS reaching up to 1200 MPa [13]. Restorations produced from 3Y-TZP require veneering over the opaque framework to achieve satisfactory esthetics; however, their most significant drawback is the frequent occurrence of chipping. To address this issue, the current trend is to use translucent, multilayered, strength-gradient zirconia either as monolithic restorations or with selective veneering [13,14]. Monolithic zirconia restorations exhibit relatively low fracture rates in short-term assessments; however, these outcomes are largely determined by the material’s microstructural characteristics [7,15].

Uniaxial and biaxial flexural testing methods are commonly utilized to assess the mechanical strength of ceramic materials. FS can be assessed using three-point, four-point, uniaxial, or biaxial flexure tests, all of which involve applying a static load to the specimen until fracture occurs [16].

The aim of this study is to compare the translucency parameters (TPs) and FS of three types of zirconia with distinct microstructural characteristics and to elucidate the relationship between these properties. The null hypothesis states that there is no significant difference in translucency or FS among the three zirconia materials investigated.

## Materials and methods

Sample preparation procedure

**Figure 1:**
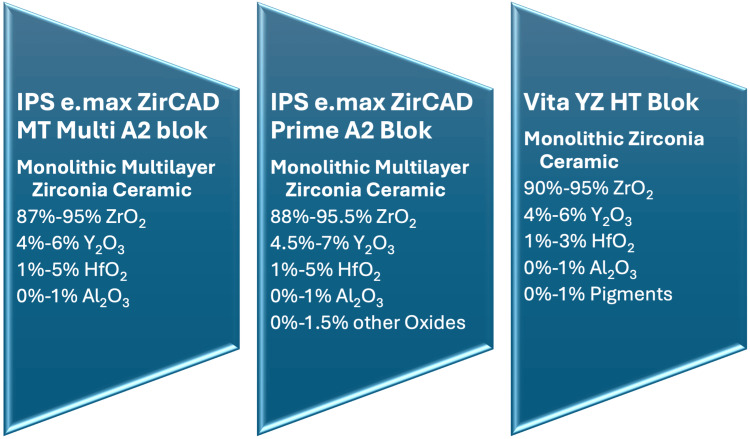
Materials used in the study, their classifications, structural characteristics ZrO_2_:Zirconium dioxide; Y_2_0_3_:Yttrium(III) oxide; HfO_2_:Hafnium(IV) oxide; Al_2_O_3_ :Aluminium oxide

Zirconia specimens in this study were prepared using IPS e.max ZirCAD Prime A2 (Ivoclar Vivadent, Schaan, Liechtenstein), Vita YZ HT (Vita Zahnfabrik, Baden-Württemberg, Germany), and IPS e.max ZirCAD MT Multi A2 (Ivoclar Vivadent) ceramic blocks. These materials were classified based on their type and structure, as shown in Figure 1. The specimens were designed in Standard Tessellation Language (STL) format with a 20% shrinkage adjustment for sintering. The zirconia specimens were detached from their connection points, sequentially polished with 600-, 800-, and 1000-grit silicon carbide abrasive papers (English Abrasive and Chemicals Ltd., London, UK), and ultrasonically cleaned at 37°C for 15 minutes to eliminate surface residues. After sintering according to the manufacturer’s instructions, the specimens’ thickness was measured with a digital caliper, confirming an anticipated shrinkage of approximately 20%. The final dimensions of the specimens prepared for FS and TP testing were measured as 14 mm × 12 mm × 2 mm.

Color measurement

Baseline color values were measured using a spectrophotometer (Vita Easyshade 4.0, Bad Säckingen, Waldshut, Germany) based on the CIE L*a*b* color system (Commission Internationale de l'Éclairage, Vienna, Austria), with calibration performed before each measurement according to the manufacturer’s instructions. All color and translucency evaluations were carried out by a single blinded operator at a consistent time of day to ensure measurement reliability. Translucency was assessed using black (b) and white (w) backgrounds. During all measurements, the spectrophotometer was consistently positioned perpendicular to the specimen surface to ensure reproducibility. The TPs (L*, a*, b*) of the samples were measured and calculated using the CIEDE2000 color difference formula.

The CIE established a color measurement system in which L* represents the lightness axis, a* corresponds to the green-red axis (negative a* indicating green and positive a* indicating red), and b* denotes the blue-yellow axis (negative b* indicating blue and positive b* indicating yellow). Translucency was assessed using white (w) and black (b) backgrounds, and the TP was calculated using the following formula [12].

$TP = \sqrt{(L_b - L_w)^2 + (a_b - a_w)^2 + (b_b - b_w)^2}$

Uniaxial FS analysis

The FSs of the zirconia bars were determined using a three-point bending test performed in a universal testing machine (Model DVT GPE; İstanbul, Türkiye) at a crosshead speed of 1 mm/min with a load capacity of 5 KN. The maximum load causing specimen fracture was recorded in Newtons (N). The mean fracture strength (MPa) was then calculated according to the ISO 6872 standard using the following equation:

$\text{Fracture strength (MPa)} = \frac{3NL}{2bd^2}$ where N is the fracture load (N), L is the specimen length (mm), b is the specimen width (mm), and d is the specimen thickness (mm). 

Statistical analysis

The data obtained from the study were transferred to a computer and analyzed using IBM SPSS Statistics software, version 25.0 (IBM Corp., Armonk, NY). In descriptive analyses, frequency data were presented as numbers (n) and percentage (%), while numerical data were expressed as means ± standard deviation and medians (Q1-Q3). The Kruskal-Wallis test was used for comparisons among more than two independent groups, and when a significant difference was detected, post hoc Mann-Whitney U tests with Bonferroni correction were performed. The Spearman correlation test was employed to evaluate the relationship between two numerical variables that did not follow a normal distribution. Correlation coefficient (r) values were interpreted as negligible for 0.00-0.19, low for 0.20-0.39, moderate for 0.40-0.69, high for 0.70-0.89, and very high for 0.90-1.00. For all statistical analyses, a significance level of p < 0.05 was considered.

## Results

**Figure 2:**
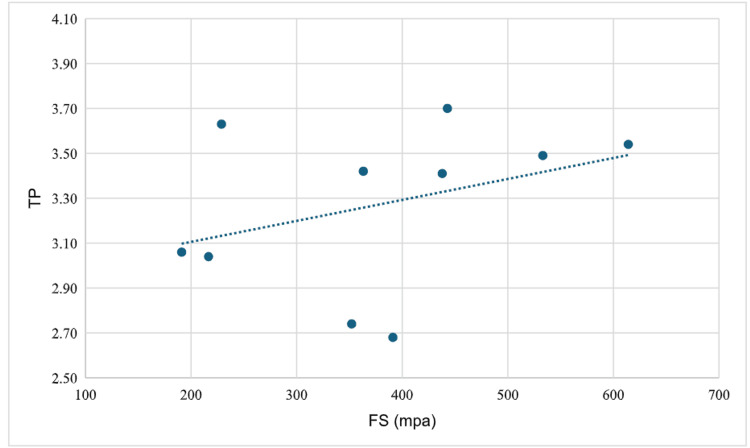
Correlation between FS and TP for IPS e.max ZirCAD MT FS: flexural strength; TP: translucency parameter; MPa: megapascal

**Figure 3:**
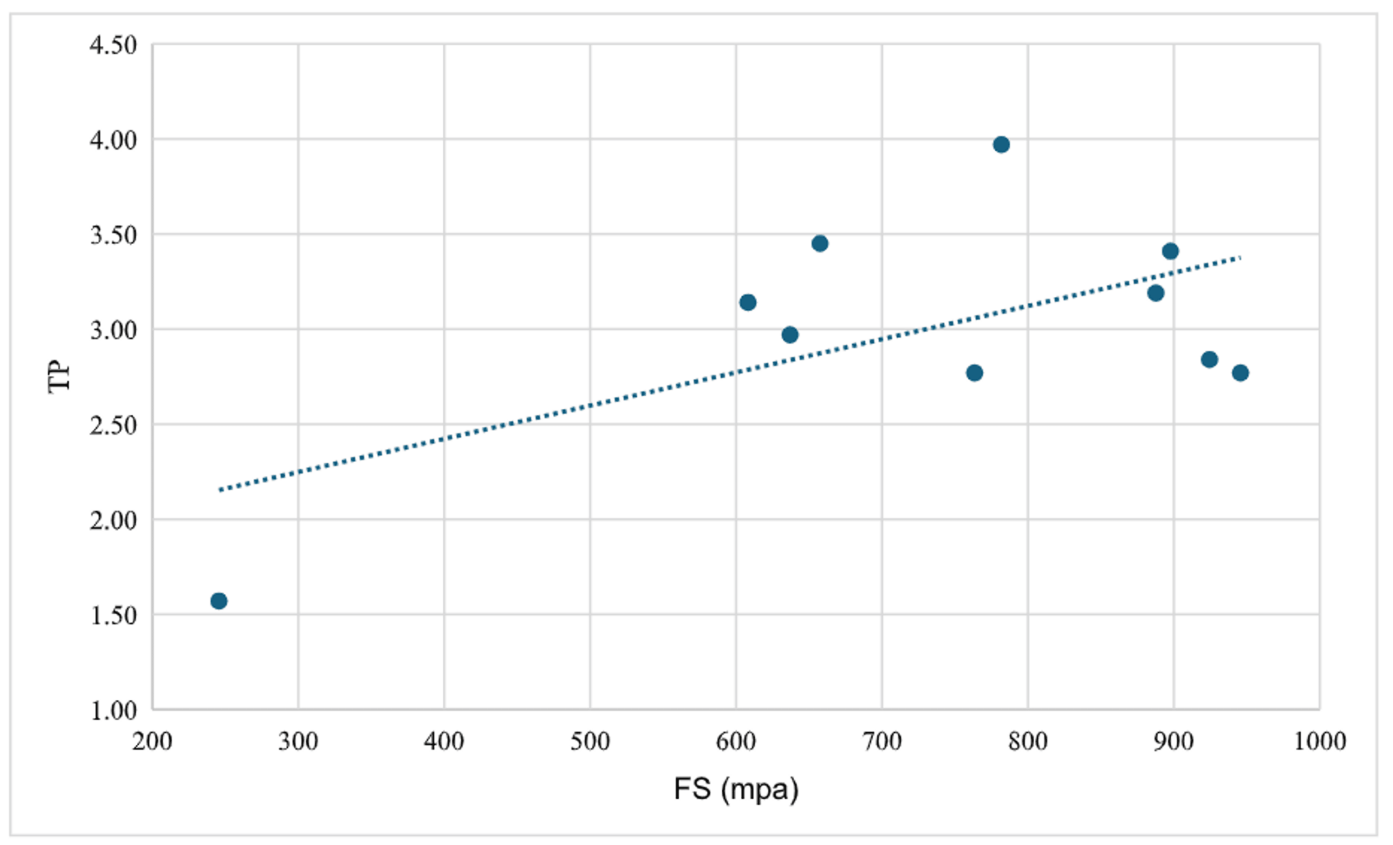
Correlation between FS and TP for VITA YZ HT FS: flexural strength; TP: translucency parameter; MPa: megapascal

**Figure 4:**
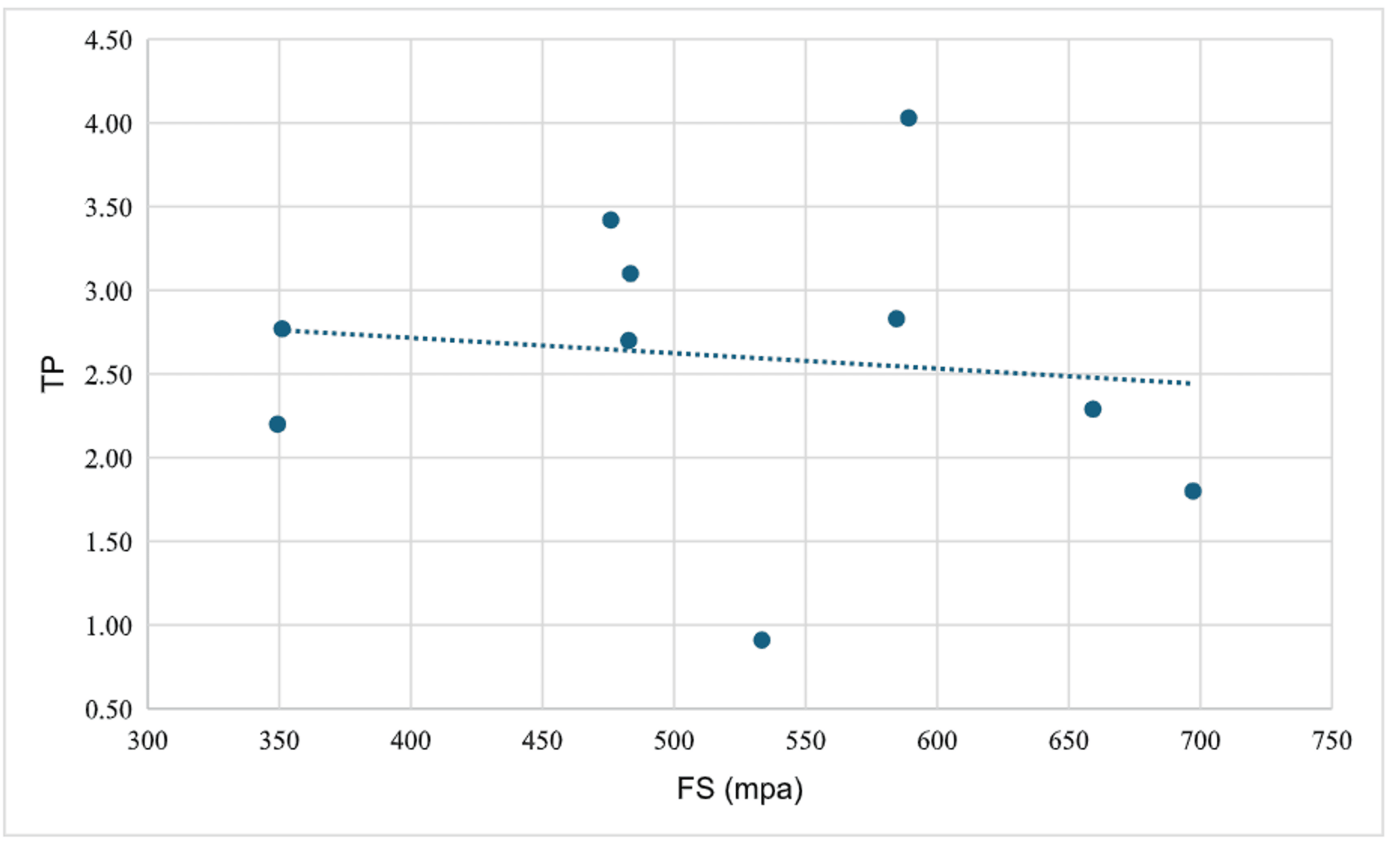
Correlation between FS and TP for IPS e.max ZirCAD Prime FS: flexural strength; TP: translucency parameter; MPa: megapascal

The FS and TP values of the materials used in the study are presented in Table 1. When the FS values were compared according to the materials used, a statistically significant difference was found (p = 0.001). Post hoc comparisons revealed that this difference originated from the higher FS values of the VITA YZ HT group compared with the other two groups. When the TP values were compared according to the materials used, no statistically significant difference was observed (p = 0.119) (Table 2). The correlation between FS and TP for IPS e.max ZirCAD was found to be not statistically significant (r = 0.055, p = 0.881) (Figure 2). The correlation between FS and TP for VITA was found to be not statistically significant (r = -0.321, p = 0.365) (Figure 3). The correlation between FS and TP for IPS e.max ZirCAD Prime was found to be not statistically significant (r = 0.018, p = 0.960) (Figure 4).

**Table 1:** FS and TP values of the materials used in the study FS: flexural strength; TP: translucency parameter; MPa: megapascal, SD: standard deviation; Q1-Q3: median

Variable	Mean±SD	Median	Q1-Q3
FS (MPa)	544.19±215.52	533.23	360.36-668.67
TP	2.96±0.68	3.05	2.72-3.42

**Table 2:** Comparison of FS and TP values according to the materials used FS: flexural strength; TP: translucency parameter; MPa: megapascal, SD: standard deviation; Q1-Q3: median The Kruskal–Wallis test was used. A significance level of p < 0.05 was considered.

	Material	Median	Q1-Q3	Mean±SD	H	p
FS (MPa)	IPS emax ZirCAD MT	377.13	225.74-465.34	377.06±138.21	13.659	0.001
	VITA YZ HT	772.62	629.79-904.42	734.91±211.93		
	IPS emax ZirCAD Prime	508.8	444.76-606.67	520.59±116.53		
TP	IPS emax ZirCAD MT	3.41	2.96-3.56	3.27±0.36	4.250	0.119
	VITA YZ HT	3.05	2.77-3.41	3.00±0.62		
	IPS emax ZirCAD Prime	2.73	2.10-3.8	2.60±0.86		

## Discussion

The aim of this in vitro study was to evaluate whether zirconia ceramics with different microstructures exhibit differences in translucency and FS. According to the results obtained, the null hypothesis was partially rejected. Differences in FS and translucency were observed among all zirconia ceramics; however, the differences in translucency were not statistically significant, while the differences in FS were significant.

Y-TZP is one of the most widely used materials in dental prosthetics due to its high FS, fracture toughness, and elastic modulus [13]. Typically stabilized with 3 mol% Y₂O₃, Y-TZP has recently evolved into multilayer forms designed to combine the advantages of different yttria contents within a single material [14]. The FS of multilayer Y-TZP depends on factors such as specimen dimensions, layer distribution, and the layer subjected to maximum tensile stress during loading. Although previous research has investigated individual layers, accurately determining the FS of multilayer zirconia remains challenging but clinically significant [17]. The three-point bending test is commonly used to measure the FS of rectangular brittle materials [18]. Specimen thickness, material homogeneity, porosity, loading contact area, and loading speed are considered important test parameters [19].

In general, the data obtained in such studies are influenced by a variety of factors, including the experimental design, testing conditions, and applied methodologies. Milling and surface preparation, in particular, have a substantial impact on the FS of ceramic materials, as these processes can introduce surface flaws that compromise the material’s integrity even before testing [20-22]. The findings of the present study demonstrated that Vita YZ HT exhibited the highest FS, followed by IPS e.max ZirCAD Prime and IPS e.max ZirCAD MT. Jerman et al. compared 3Y-TZP, 4Y-TZP, and 5Y-TZP zirconia blocks and found the highest FS in the 3Y-TZP blocks and the lowest in the 5Y-TZP blocks, which is consistent with the results of our study [23]. Contrary to our findings, Omar et al. reported a statistically significant difference in the FS of zirconia materials with different microstructures (4Y and 3-5Y). However, their observation that 3-5Y zirconia exhibited higher FS than 4Y zirconia is in parallel with the results of our study [4]. In the study by Nassary et al., six zirconia materials with different yttria contents were compared with lithium-based ceramics. They reported no statistically significant differences in FS among the zirconia materials [3]. Despite the absence of significant differences, the overall trend observed in our study supports previous research indicating that a higher zirconium dioxide (ZrO₂) phase content is positively correlated with increased FS in Y-TZP ceramics [20]. Conversely, an increased concentration of Y₂O₃, commonly employed as a stabilizing oxide, promotes the formation of a higher proportion of the cubic phase, which has been shown to reduce the FS of zirconia. Taken together, these findings suggest that both ZrO₂ content and the relative proportion of the tetragonal phase play a critical role in enhancing the mechanical performance of zirconia ceramics, particularly in terms of FS [21].

Translucency is a crucial factor in achieving esthetics in ceramic materials. In recent years, highly translucent, fully stabilized cubic/tetragonal zirconia materials have been developed, in which the cubic phase content is increased by adding greater amounts of stabilizing oxides. The presence of larger and more isotropic cubic crystals in these materials enhances translucency while preserving their high mechanical strength [3]. In the present study, three different zirconia blocks were evaluated, and although IPS e.max ZirCAD MT demonstrated the highest translucency, no statistically significant differences were observed among the materials. Consistent with these results, Ziyad et al. also reported no statistically significant differences in their study involving zirconia blocks with varying yttria contents [24]. In a study conducted by Matis et al., materials similar to those used in our research (IPS e.max ZirCAD MT Multi and IPS e.max ZirCAD Prime) were utilized. Zirconia ceramics with different thicknesses and structures, as well as lithium disilicate-based ceramics, were included in the study. According to the results, no statistically significant difference in translucency was found among the four zirconia materials of the same thickness, except for Katana STML [25]. Wang et al. conducted a study comparing the translucency of three structurally different zirconia ceramics with that of a lithium disilicate ceramic. Although the lithium disilicate ceramic demonstrated higher translucency than all zirconia ceramics, consistent with the present study, no statistically significant differences were observed among the zirconia ceramics despite their structural variations [26].

This study has several limitations. First, its in vitro design cannot fully reproduce intraoral moisture, temperature fluctuations, biofilm, and complex multiaxial loading. Second, only three zirconia formulations at a single thickness (2 mm) were tested, limiting generalizability; thickness- and material-dependent interactions remain unknown. Third, no artificial aging was applied (e.g., thermocycling, hydrothermal low-temperature degradation), and no cyclic/fatigue loading was performed, restricting inferences about time-dependent performance. Fourth, the property set was limited to FS and translucency; other clinically relevant metrics, fracture toughness, fatigue endurance limit, long-term color stability, susceptibility to low temperature degradation, and Weibull reliability, were not evaluated. Finally, microstructural parameters (grain size, phase fractions, and porosity) were not quantified, constraining the mechanistic interpretation of structure-property relationships.

## Conclusions

Within the limitations of this in vitro study, zirconia ceramics with different microstructures showed no statistically significant differences in translucency; however, significant differences were observed in FS. Vita YZ HT demonstrated significantly higher FS compared with the other zirconia ceramics, while IPS e.max ZirCAD MT exhibited the highest translucency values, though not statistically significant. These findings indicate that increasing the yttria content enhances translucency through greater cubic phase formation but may compromise mechanical performance, whereas a higher tetragonal phase fraction contributes positively to FS. Despite their lower translucency compared with lithium disilicate ceramics, zirconia materials continue to offer satisfactory optical and mechanical properties for clinical applications. Further studies involving larger sample sizes, different thicknesses, aging procedures, and long-term in vivo evaluations are recommended to better elucidate the structure-property relationships of next-generation zirconia ceramics.
